# Supplementary material for: Radiomics and Delta-Radiomics Signatures to Predict Response and Survival in Patients with Non-Small-Cell Lung Cancer Treated with Immune Checkpoint Inhibitors
Source: Cancers (Basel). 2023 Mar 25;15(7):1968. doi: 10.3390/cancers15071968 (PMC10093736; doi:10.3390/cancers15071968)
Supplement: Supplementary file 1 [file cancers-15-01968-s001.zip › Supplementary Table S1.pdf]

Supplementary Table S1: Anatomical location of the segmented lesions

Lesion location	Training set		Test set	
	Target lesions (n = 345)	Index lesions (n = 146)	Target lesions (n = 91)	Index lesions (n = 42)
Lung	173	120	47	34
Lymph node	104	14	24	2
Liver	21	4	4	2
Adrenal	24	2	9	1
Soft tissue	9	3	6	3
Other[a]	14	3	1	0

[a]Other include spleen, kidney and pleura
