# Supplementary material for: Radiomics and Delta-Radiomics Signatures to Predict Response and Survival in Patients with Non-Small-Cell Lung Cancer Treated with Immune Checkpoint Inhibitors
Source: Cancers (Basel). 2023 Mar 25;15(7):1968. doi: 10.3390/cancers15071968 (PMC10093736; doi:10.3390/cancers15071968)
Supplement: Supplementary file 1 [file cancers-15-01968-s001.zip › Supplementary Table S2.pdf]

(a)

Predictors	Relevance
IH_MedianD	6.27
Shape_Flatness	0.75
IH_Skewness	0.57
NGLDM_DE	4.57
Stats_P90	0.87
NGTDM_Coarseness	2.21
GLDZM_SDE	0.75
GLCM_ClusShade	0.71
NGLDM_EN2	4.57
Shape_VolumeDensityBE	0.28

(b)

Predictors	Relative importance	Coefficient
IH_MedianD	0.414915	0.867104
NGLDM_DE	0.275619	4.120108
Stats_P90	0.216907	-2.1779
GLCM_ClusShade	0.092559	-0.07551

(c)

Predictors	Relevance	Predictors	Relevance
Shape_Flatness	1.21	GLDZM_HIE	0.51
IH_MeanD	0.23	Shape_Sphericity	0.57
Shape_CentroidDistance	0.55	Stats_Cov	0.47
Shape_AreaDensityBB	0.63	IH_P10	0.33
LocInt_PeakGlobal	0.65	GLSZM_LIE	0.41
GLRLM_RLV	0.41	LocInt_PeakLocal	0.59
GLRLM_RE	0.51	Stats_IQR	0.26
GLCM_Energy	0.34	Shape_MajorAxisLength	0.60
Stats_MeanD	0.22	NGTDM_Contrast	0.37
NGTDM_Busyness	0.49	GLCM_InvDiffMomNor	0.41
Stats_Median	0.61	GLRLM_LGRE	0.35
Stats_Std	0.24	Stats_Kurtosis	1.03
Stats_QCOD	0.56	Stats_MedianD	0.26
Stats_P90	0.54	GLCM_Homogeneity2	0.32
GLSZM_SAE	0.48	GLCM_DiffEntro	0.34
GLCM_MaxProb	0.38	NGLDM_LGSDE	0.38
Stats_Mean	0.49	NGLDM_DNN	0.46
GLCM_SumSquares	0.26	Shape_LeastAxisLength	0.47
GLCM_Entrop2	0.28	GLSZM_SZV	0.37
GLSZM_ZP	0.28	GLCM_Homogeneity1	0.32
GLDZM_LILDE	0.58	GLCM_Dissimilar	0.33
GLSZM_LAE	0.36	GLCM_InverseVar	0.26
NGLDM_LGLDE	0.33	GLDZM_LISDE	0.43
GLRLM_LRLGE	0.41	IH_Kurtosis	1.04
GLSZM_LISAE	0.35	GLCM_DiffAvrg	0.32
