# Supplementary material for: Radiomics and Delta-Radiomics Signatures to Predict Response and Survival in Patients with Non-Small-Cell Lung Cancer Treated with Immune Checkpoint Inhibitors
Source: Cancers (Basel). 2023 Mar 25;15(7):1968. doi: 10.3390/cancers15071968 (PMC10093736; doi:10.3390/cancers15071968)
Supplement: Supplementary file 1 [file cancers-15-01968-s001.zip › Supplementary Table S3.pdf]

(a)

Predictors	Relevance
GLCM_InfoCorr1	11.79
IH_Kurtosis	2.55
NGLDM_DV	2.91
Stats_P90	1.19
IH_MinGradI	0.72
GLSZM_SAE	2.47
GLCM_InfoCorr2	6.20
IH_MedianD	4.72
NGTDM_Coarseness	0.44
NGLDM_LGSDE	0.85

(b)

Predictors	Relative importance	Coefficient
GLCM_InfoCorr1	0.20	-11.31
NGLDM_DV	0.28	1.29
GLCM_InfoCorr2	0.24	-7.07
IH_MedianD	0.27	1.41

(c)

Predictors	Relevance	Predictors	Relevance
NGLDM_DV	2.13	LocInt_PeakLocal	1.42
IH_Mode	0.46	GLRLM_GLNN	0.80
GLSZM_LILAE	0.91	Stats_RMS	1.34
GLCM_ClusProm	1.03	IH_Entropy	0.77
GLCM_Entrop2	0.74	GLCM_Energy	1.07
GLRLM_SRE	0.93	Stats_IQR	0.70
Shape_MaxDiameter_3_D	0.83	GLCM_Homogeneity1	0.84
Stats_Cov	1.53	IH_MedianD	0.17
NGLDM_DNN	1.60	GLCM_DiffEntro	0.84
Shape_Flatness	1.95	GLDZM_LILDE	1.24
NGLDM_GLN	0.74	GLCM_Homogeneity2	0.92
Stats_QCOD	1.54	NGLDM_LGLDE	0.77
NGTDM_Busyness	1.42	GLCM_Dissimilar	0.92
GLRLM_RP	1.05	IH_Cov	1.23
Shape_VolumeDensityBB	1.56	GLRLM_LRLGE	0.80
GLCM_Correl1	1.54	GLSZM_LISAE	0.93
Shape_CentroidDistance	1.47	Shape_MajorAxisLength	1.02
Shape_VolumeDensityBE	1.82	IH_Kurtosis	1.57
LocInt_PeakGlobal	1.74	IH_Uniformity	0.84
NGTDM_Contrast	1.12	GLDZM_LISDE	1.04
Shape_Compactness3	1.23	NGLDM_LGSDE	1.38
GLCM_InvDiffMomNor	1.14	GLDZM_HISDE	1.18
NGLDM_LDE	1.45	NGTDM_Coarseness	0.77
GLCM_MaxProb	1.21	GLSZM_IN	0.96
GLCM_Contrast	0.78	NGLDM_GLNN	0.82
