# Supplementary material for: Radiomics and Delta-Radiomics Signatures to Predict Response and Survival in Patients with Non-Small-Cell Lung Cancer Treated with Immune Checkpoint Inhibitors
Source: Cancers (Basel). 2023 Mar 25;15(7):1968. doi: 10.3390/cancers15071968 (PMC10093736; doi:10.3390/cancers15071968)
Supplement: Supplementary file 1 [file cancers-15-01968-s001.zip › Supplementary Table S4.pdf]

(a)

Predictors	Relevance
Shape_MinorAxisLength_delta	43.54
GLCM_Entrop2	1.28
GLDZM_HISDE_delta	1.87
Stats_Cov_delta	1.11
Shape_Compactness2_delta	0.08
IH_MinGrad_delta	7.23
GLCM_Correl1_delta	9.72
GLCM_InfoCorr1	5.04
IH_MedianD	3.94
GLDZM_SDE_delta	8.43

(b)

Predictors	Relative importance	Coefficient
Shape_MinorAxisLength_delta	0.46	-7.79
GLCM_Entrop2	0.11	-4.25
IH_MinGrad_delta	0.12	2.98
GLCM_InfoCorr1	0.17	-12.81
IH_MedianD	0.14	1.90

(c)

Predictors	Relevance	Predictors	Relevance
Shape_Compactness_delta	1.57	NGTDM_Busyness_delta	0.18
Shape_VolumeDensityBE	0.21	IH_P10	0.07
IH_QCOD	0.11	GLCM_Correl1_delta	0.38
Stats_P10_delta	0.11	GLSZM_SZNN_delta	0.26
Shape_Sphericity_delta	0.16	GLCM_Correl1	0.17
IH_Skewness_delta	0.27	IH_QCOD_delta	0.09
IH_P90_delta	0.08	Shape_CentroidDistance	0.12
GLSZM_SAE_delta	0.26	GLSZM_SZV_delta	0.53
GLRLM_RLNN_delta	0.13	IH_Median	0.05
Shape_AreaDensityBE	0.16	GLSZM_ZP_delta	0.27
GLRLM_GLN_delta	0.47	NGLDM_EN2	0.24
NGLDM_SM	0.17	NGLDM_LGLDE	0.06
Fractal_Average_delta	0.12	GLDZM_HISDE_delta	0.10
GLDZM_SDE	0.20	GLCM_InvDiffNorm_delta	0.46
IH_MinGrad_delta	0.41	IH_P90	0.07
NGTDM_Contrast_delta	0.20	Stats_Kurtosis_delta	0.11
Stats_Max_delta	0.15	Stats_Mean_delta	0.18
GLCM_ClusShade	0.16	NGLDM_DNN_delta	0.09
NGLDM_SM2	0.30	NGTDM_Strength_delta	0.12
LocInt_PeakGlobal_delta	0.39	Fractal_Lacunarity	0.13
GLDZM_LDE	0.18	Shape_Elongation_delta	0.20
IH_Kurtosis	0.18	GLSZM_LAE_delta	0.56
GLCM_InvDiffMomNor_delta	0.54	Shape_MajorAxisLength_delta	1.37
Shape_AreaDensityBE_delta	0.26	GLSZM_LILAE_delta	0.32
GLDZM_ZP_delta	0.28	Stats_Mean	0.15
