# Supplementary material for: Radiomics and Delta-Radiomics Signatures to Predict Response and Survival in Patients with Non-Small-Cell Lung Cancer Treated with Immune Checkpoint Inhibitors
Source: Cancers (Basel). 2023 Mar 25;15(7):1968. doi: 10.3390/cancers15071968 (PMC10093736; doi:10.3390/cancers15071968)
Supplement: Supplementary file 1 [file cancers-15-01968-s001.zip › Supplementary Table S5.pdf]

(a)

Predictors	Relevance
Stats_Max	3.72
Stats_P10	0.66
Shape_Elongation	0.93
Stats_Cov	0.35
IH_Mode	0.62
Fractal_Lacunarity	0.51
Stats_Median	0.81
GLRLM_GLNN	0.6
NGLDM_DE	1.8
GLSZM_SAE	0.38

(b)

Predictors	Relative importance	Coefficient
Stats_Max	0.51	0.65
Shape_Elongation	0.17	1.04
IH_Mode	0.32	-0.23

(c)

Predictors	Relevance	Predictors	Relevance
Shape_Elongation	2.00	Stats_MedianD	0.34
GLCM_SumAvg	0.42	GLRLM_RLV	0.47
LocInt_PeakGlobal	0.89	LocInt_PeakLocal	1.19
Stats_P90	1.10	GLCM_MaxProb	0.55
Fractal_SD	1.04	GLCM_Contrast	0.47
Stats_Mean	1.14	GLCM_DiffEntro	0.42
GLRLM_RE	0.91	GLSZM_LILAE	0.72
GLDZM_ZP	0.48	GLCM_Energy	0.46
Shape_LeastAxisLength	0.70	GLRLM_GLNN	0.55
Stats_Cov	0.83	GLCM_DiffVar	0.52
NGLDM_DV	0.64	NGLDM_LGLDE	0.56
IH_RMeanD	0.58	Stats_RMeanD	0.57
GLRLM_SRE	0.45	IH_MedianD	0.10
Shape_Asphericity	0.71	IH_Cov	0.56
GLDZM_DZV	0.75	Shape_Sphericity	0.70
Stats_Median	1.24	GLCM_InvDiffMomNor	0.70
Shape_VolumeDensityBB	1.48	GLSZM_ZP	0.46
GLCM_Homogeneity2	0.43	NGLDM_DNN	0.50
IH_Entropy	0.49	NGLDM_GLN	0.44
Stats_IQR	0.39	Shape_AreaDensityBB	1.68
GLDZM_LILDE	0.78	Shape_Compactness3	0.70
NGLDM_LDE	0.46	Stats_Kurtosis	0.78
NGTDM_Busyness	0.93	Shape_CentroidDistance	1.29
Stats_QCOD	0.97	Shape_Compactness2	0.73
GLCM_Entrop2	0.51	Shape_AreaDensityBE	0.91
