# Supplementary material for: Radiomics and Delta-Radiomics Signatures to Predict Response and Survival in Patients with Non-Small-Cell Lung Cancer Treated with Immune Checkpoint Inhibitors
Source: Cancers (Basel). 2023 Mar 25;15(7):1968. doi: 10.3390/cancers15071968 (PMC10093736; doi:10.3390/cancers15071968)
Supplement: Supplementary file 1 [file cancers-15-01968-s001.zip › Supplementary Table S6.pdf]

(a)

Predictors	Relevance
NGLDM_DE	5.74
NGLDM_SM	0.78
Shape_Flatness	0.6
Stats_P90	2.63
Shape_CentroidDistance	1.22
GLRLM_RLV	4.82
GLRLM_GLV	0.15
GLCM_Energy	0.72
GLCM_SumEntro	0.21
NGTDM_Busyness	0.16

(b)

Predictors	Relative importance	Coefficient
NGLDM_DE	0.73	-2.90
Shape_Flatness	0.27	-0.68

(c)

Predictors	Relevance	Predictors	Relevance
Shape_Elongation	3.55	Shape_CentroidDistance	2.25
IH_Kurtosis	1.97	GLCM_Contrast	1.24
GLCM_DiffVar	1.34	IH_MedianD	0.20
Shape_MaxDiameter_2_D0	1.64	Stats_QCOD	2.19
GLCM_Homogeneity2	1.22	GLCM_Homogeneity1	1.21
GLSZM_SAE	1.94	GLDZM_LILDE	1.72
Stats_Cov	1.93	GLCM_Entrop2	1.11
GLCM_DiffAvrg	1.14	GLCM_MaxProb	1.40
GLRLM_RLV	1.48	GLCM_Dissimilar	1.11
Shape_VolumeDensityBB	2.74	GLCM_Energy	1.21
NGTDM_Contrast	1.57	GLCM_DiffEntro	1.15
GLCM_InvDiffMomNor	1.49	GLCM_InverseVar	1.35
GLSZM_ZP	1.45	Stats_MedianD	0.70
NGLDM_LDE	1.43	Shape_Asphericity	1.78
LocInt_PeakGlobal	2.63	GLDZM_ZP	1.43
Stats_Median	2.59	NGLDM_DNN	1.63
Stats_RMS	2.29	GLSZM_SZV	1.44
Shape_SpherDisprop	1.91	GLSZM_LILAE	1.56
GLRLM_GLNN	1.04	NGLDM_SDE	1.37
GLCM_SumSquares	0.92	GLRLM_LRE	1.36
Shape_VolumeDensityBE	2.41	Shape_Flatness	3.00
Stats_RMeanD	1.09	Shape_AreaDensityBB	3.10
Stats_P90	2.44	NGTDM_Strength	1.82
LocInt_PeakLocal	3.16	GLSZM_LAE	1.42
NGTDM_Busyness	1.98	GLSZM_SZNN	1.87
