# Supplementary material for: Radiomics and Delta-Radiomics Signatures to Predict Response and Survival in Patients with Non-Small-Cell Lung Cancer Treated with Immune Checkpoint Inhibitors
Source: Cancers (Basel). 2023 Mar 25;15(7):1968. doi: 10.3390/cancers15071968 (PMC10093736; doi:10.3390/cancers15071968)
Supplement: Supplementary file 1 [file cancers-15-01968-s001.zip › Supplementary Table S7.pdf]

(a)

Predictors	Relevance
Shape_Volume_delta	26.5
GLDZM_LILDE_delta	22.57
IH_P10	0.52
Shape_Sphericity_delta	1.17
Fractal_Average_delta	1.09
Stats_P90	2.65
Shape_AreaDensityBE_delta	8.08
NGTDM_Contrast_delta	3.26
IH_Energy_delta	12.41
GLCM_Dissimilar_delta	4.16

(b)

Predictors	Relative importance	Coefficient
Shape_Volume_delta	0.56	0.76
GLDZM_LILDE_delta	0.44	4.61

(c)

Predictors	Relevance	Predictors	Relevance
Shape_Volume_delta	1.55	Stats_QCOD	0.24
GLSZM_IV	0.20	GLRLM_GLV	0.20
GLCM_AutoCorr_delta	0.18	Shape_AreaDensityBE_delta	0.58
Shape_VolumeDensityBB	0.67	IH_RMeanD	0.22
GLRLM_SRLGE	0.14	IH_Skewness_delta	0.32
GLSZM_SZNN_delta	0.44	Stats_Cov	0.32
NGLDM_DV	0.29	LocInt_PeakLocal	0.43
LocInt_PeakGlobal_delta	0.60	GLSZM_SZNN	0.22
Shape_Compactness2_delta	0.29	GLCM_InvDiffNorm_delta	0.32
Stats_RMeanD_delta	0.21	GLSZM_ZP_delta	0.31
Shape_Elongation_delta	0.69	GLSZM_LAE_delta	0.41
IH_QCOD_delta	0.30	IH_IQR	0.07
Stats_RMS	0.24	Shape_MajorAxisLength	0.28
GLCM_InvDiffMomNor	0.22	NGTDM_Contrast_delta	0.29
GLSZM_INN	0.23	GLCM_InvDiffMomNor_delta	0.48
Shape_Asphericity	0.37	Fractal_SD_delta	0.37
IH_MinGrad_delta	0.54	GLCM_MaxCorr_delta	0.23
GLRLM_GLN_delta	0.41	GLRLM_RP_delta	0.25
IH_Kurtosis	0.27	Stats_Median	0.33
GLDZM_LILDE_delta	1.04	NGTDM_Busyness	0.31
GLSZM_HIE	0.16	Stats_Kurtosis_delta	0.26
Shape_Elongation	0.91	NGLDM_DV_delta	0.29
IH_MaxGradI_delta	0.30	GLRLM_LRE_delta	0.25
GLCM_ClusProm	0.20	GLSZM_LILAE_delta	0.30
GLDZM_ZP_delta	0.31	Stats_Median_delta	0.28
